# Chemical composition, mineral profile, anti-bacterial, and wound healing properties of snail slime of *Helix aspersa* Müller

**DOI:** 10.37796/2211-8039.1424

**Published:** 2023-12-01

**Authors:** Marouane Aouji, Amine Rkhaila, Bouchra Bouhaddioui, Malak Zirari, Hala Harifi, Youness Taboz, Lalla Aicha Lrhorfi, Rachid Bengueddour

**Affiliations:** a Laboratory of Natural Resources and Sustainable Development, Department of Biology, Faculty of Sciences, Ibn Tofail University, BP 133, Kenitra 14000, Morocco; b Laboratory of Plant, Animal and Agro-Industry Productions, Department of Biology, Faculty of Sciences, Ibn Tofail University, BP 133, Kenitra 14000, Morocco; c Laboratory of Organic Chemistry Catalysis and Environment, Department of Chemistry, Faculty of Sciences, Ibn Tofail University, BP 133, Kenitra 14000, Morocco; d Laboratory of Biology and Health, Department of Biology, Faculty of Sciences, Ibn Tofail University, BP 133, Kenitra 14000, Morocco

**Keywords:** Activity antibacterial, Chemical composition, *H. aspersa* Müller, Slime, Wound healing

## Abstract

Mucus is a substance made by snails that serves a variety of purposes and is increasingly employed in the medical and cosmetic industries. It includes bioactive compounds with a range of biological characteristics that could be useful in the treatment of particular issues. This study assessed the wound-healing efficiency, antibacterial activity, chemical and mineral composition of *Helix aspersa* Müller slime. Inductively Coupled Argon Plasma Atomic Emission Spectrometry (ICP-OES) was used for mineral analysis, while Gas chromatography coupled to mass spectrometry (GC–MS) analysis was used for chemical characterization. The findings showed that the *H. aspersa* Müller slime had inhibitory activity on the test samples. Additionally, it revealed significant healing activity. These findings point to the chemical composition and various biological activities of the *H. aspersa* Müller slime, which may be related to the animal’s particular functions and be useful for medical applications. Our findings suggest that the *H. aspersa* Müller slime has biological effects related to antimicrobial activities and wound healing, and they pave the way for a more thorough investigation of its potential therapeutic effects.

## 1. Introduction

Due to the serious public health issue of antibiotic resistance, researchers and medical professionals must find new, efficient antimicrobial agents [1,2]. According to the World Health Organization, *Acinetobacter baumannii*, *Pseudomonas aeruginosa*, and several species of the *Enterobacteriaceae* family are given worldwide priority when it comes to the discovery of novel antimicrobial medicines for these antibiotic-resistant bacteria [3].

Invertebrates only have an innate immune system to fight against invading diseases since they lack the adaptive immune system present in vertebrate species. Given this group of creatures’ exceptional evolutionary success, it is obvious that their innate immune systems are quite powerful in invertebrates [4]. This finding has led to extensive studies of several invertebrate species in recent years. Most of the antimicrobial peptides present in invertebrate hemolymph are effective against a variety of microorganisms [5–7].

For millennia, people have consumed land snails and employed them in a variety of medicinal procedures [8,9]. Snails utilize slime for various functions, serving as a defense mechanism, lubricant, emollient, adhesive, and for hydration [10,11]. This slime has found applications in the human health and cosmetics industries [12–14]. Numerous biological activities, including antibacterial, antioxidant, anti-tyrosinase, and antitumor ones, have been linked to snail slime [13,15,16]. Snail mucus has also been shown to include a wide range of substances, including allantoin, hyaluronic acid, peptides, and proteins [8,10,17].

Additionally, snails have the capacity to secrete huge amounts of mucin, which includes antibacterial proteins and confers some resistance to pathogen infection [18]. Moreover, several studies have demonstrated that bioactive substances obtained from various types of snail mucus may be employed in a broad variety of treatments, including lotions for the treatment of skin abrasions and scars, respiratory conditions, and heartburn [19].

When coupled with several conditions, such as wound length and depth, wound infections, and underlying disorders like diabetes, the wound is a complication that affects individuals, and treatment becomes more crucial [20].

Microorganisms typically reside on the skin’s surface, but when the skin is damaged, they might enter the deeper tissue. Based on factors including infection state, replication status, and tissue load of microorganisms, wound infection is classified as bacterial colonization, local infection, or invasive systemic infection [21].

Burns are one of the world’s major health problems, particularly in developing countries [22]. Microbial infections are considered a major problem for burn patients [23]. According to a recent study by Zlatko [24], certain resistant bacteria strains in burns may lessen the speed at which they recover. Major agents that induce wound infection include *Staphylococcus aureus* and *Pseudomonas aeruginosa* [25].

According to studies on the components of snail secretions, *Helix aspersa* slime includes a significant amount of organic compounds with advantageous and healing qualities for human skin, such as allantoin and glycolic acid [17].

The present research aimed to examine the chemical and mineral composition of the snail *H. aspersa* Müller slime, as well as its antibacterial activity and healing potential.

## 2. Material and methods

### 2.1. Extraction of H. aspersa Müller slime

Extraction was carried out manually by stimulating the pedal glands using a small sterile metal rod pointed at the end. It was not necessary to euthanize the snails, and their handling was carried out in compliance with the principles of animal welfare for scientific research.

### 2.2. GC–MS analysis of the chemical content

Gas chromatography coupled to mass spectrometry was used to evaluate extracts under the following conditions: Injector port temperature is 250 °C. The starting oven temperature is 40 °C, and the temperature is gradually increased to 8 °C.min^−1^ for 18 min until it reaches 260 °C. TheBR-5ns FS capillary column (30m×0.25mmID × 0.25m)was utilized. In undivided mode, the injection volume of heliumis 1.0 mLmin^−1^. The whole analysis took 100 min.

The mass spectrometry detector (MSD) was set to electronic impact ionization mode, with an ionizing energy of 70 eV and an *m*/*z* scan range of 50–500.

The temperature of the ion source was 230 °C, and then it quadrupled to 150 °C. With a 3-min solvent delay, the electron multiplier voltage (EM voltage) was held at 1100 V above the self-regulatory limit.

### 2.3. Total ash

According to ISO 936 [26], total ash from snail samples was evaluated using the gravimetric technique at 550 °C. The results were represented as a percentage (g of total ash per 100 g).

### 2.4. Mineral analysis

A 1g test sample was used to identify the mineral components in the calcined residue after 16 h at 480 °C. The ash was diluted in strong nitric acid (25% concentration) and then filtered. This extract was used to determine mineral composition. The trace elements studied were determined using Inductively Coupled Argon Plasma Atomic Emission Spectrometry (RF power 1500 W, plasma gas flow rate (Ar) 8 L/min, auxiliary gas flow rate (Ar) 0.2 L/min, Axial View Size, copying and Playback time of 45 min, and copying time of 15 min).

Due to the presence of phosphorus in organic molecules, oxidizing the organic material to release the phosphorus necessitates a digestion or calcination operation. The total phosphorus content of *H. aspersa* Müller flesh was then colorimetrically measured using the AOAC Method [27].

### 2.5. Antimicrobial assays

#### 2.5.1. Bacterial strains

The antibacterial properties of *H. aspersa* Müller slime have been determined against *P. aeruginosa* have been isolated and identified at Plant, Animal and Agro-Industry Productions Laboratory (PAAP lab), Ibn Tofail University, and stored at −4 °C in glycol. Other reference strains are used in this study (*L. monocytogenes* (ATCC 7644), *S. aureus* (ATCC 29213), and *E. coli* (ATCC 35218)). In order to obtain live and cultivable bacteria, 1 mL of stored bacterial suspension was mixed with 2 mL of nutrient broth.

#### 2.5.2. Antibiotics

The antibiotics used were ampicillin (AMP), cefuroxime sodium (CUS), cefotamine sodium (COF), erythromycin (ETM), netilmicin (NM), piperacillin (PRP) and tetracycline (TC) (Table 1).

#### 2.5.3. Disc diffusion method

In this study, the antimicrobial effect of *H. aspersa* Müller slime was evaluated using the Kirby Bauer disk diffusion method [28]. To assess this activity, Wathman N°3 paper disks (6 mm) were boiled for 30 min to remove any chemical that might inhibit microbial growth, sterilized, and then stored in tightly closed sterile glass vials until use. Each disk was then coated with different concentrations of slime, and antibiotic disks were placed on the surface of the MH medium, pre-inoculated by swabbing with bacterial suspensions (10^8^ CFU). The infected Petri dishes were then incubated at 37 °C in the dark. 24 h after incubation, the diameter of the inhibitory zones, measured in millimeters, was determined. The bacteria have been grouped for the purpose of utilizing the latter [29].

The results were interpreted as follows:

- Resistant: diameter ≤ 8 mm;
- Moderately sensitive: diameter between 9 and 14 mm;
- Sensitive: diameter between 15 and 19 mm;
- Extremely sensitive: diameter > 20 mm.

#### 2.5.4. Determination of the minimum inhibitory concentration (MIC)

Using the Wiegand et al. [30] method, the lowest inhibitory concentration of *H. aspersa* Müller slime against the chosen strains was calculated. 20 mL of fresh bacterial culture (1 × 10^4^ CFU) were added and inoculated onto *H. aspersa* Müller slime (10, 20, 40, 60, 80, and 100 μg·mL^−1^). Bacterial growth was detected at 600 nm after the reaction had been incubated overnight. MIC was established as the lowest sample concentration that prevented the formation of turbidity using PBS as a negative control.

### 2.6. Study of the healing activity of slime of H. aspersa Müller

The aim of this study was to assess the potential for accelerating dermal tissue neoformation after application of *H. aspersa* Müller slime to superficial scars. The comparison was made between a group of animals receiving a reference cream (Madecassol ®) and a group treated with physiological water.

The institutional ethical committee for the care and use of the laboratory animals at the Faculty of Sciences, Ibn Tofail University, kenitra 14,000, Morocco, reviewed and approved the present study and animal rights were respected.

#### 2.6.1. Rat preparation

Female Wistar albino rats weighing between 150 and 200 g (4–5 months old) underwent this assessment in vivo. Three groups of five animals each were used (each group was housed in its own cage). Group I functioned as the negative control (no treatment), Group II was given Madecassol ® as a positive control, and Group III received *H. aspersa* Müller slime as a treatment. For 28 days, wounds should be treated once per day.

#### 2.6.2. Experimental wound

Chloral was used to anesthetize the animals (0.5 mL.100 g^−1^ per kilogram of body weight) intraperitoneally. Wounds were made in accordance with Akbari et al. [31] procedures.

#### 2.6.3. Wound healing rate

Wound diameters were measured immediately after wound creation and then every 7 days after wounding. In addition, the epithelialization period was monitored for each group. In addition, the wound areas of the rats were photographed weekly over the 28 days. The length and width of the wounded areas were then measured. The area of wound contraction was then calculated according to the method of [32] using the following formula:


$$\%\;\text{of Wound closure}=\frac{\text{Day }0\;\text{Wound area}-\text{Day n Wound area}}{\text{Day }0\;\text{Wound area}}$$

Where n is the day of wound surface measurement other than day 0.

### 2.7. Statistical analyses

The results of the tests that were run are shown as a mean ± standard deviation in triplicates. One-way analysis of variance (ANOVA) and Duncan’s test were used in the statistical analysis. P ≤ 0.05 was adopted as the significance threshold.

## 3. Result and discussion

### 3.1. Chemical analysis

**Fig. 1:**
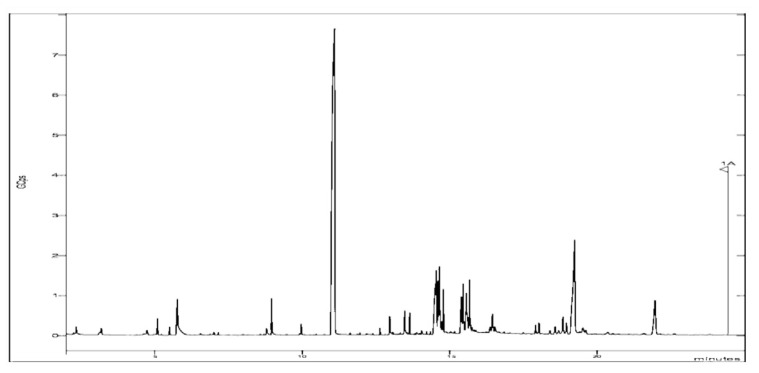
GC–MS profile of H. aspersa Müller slime.

Twenty chemical substances were identified in the *H. aspersa* Müller slime during GC–MS analysis (Table 2, Fig. 1). The volatility of the chemicals may be inferred from the total ion current time that appeared on the TIC profile. More volatile substances were thought to elute at shorter retention periods. Esters, alcohols, aldehydes, ketones, and sulfur compounds were the primary types of volatile substances discovered in *H. aspersa* Müller slime.

The most abundant were cyclotrisiloxane hexamethyl, 1,2-benzene dicarboxilic acid, octasiloxane, 1,1,3,3,5,5,7,7,9,9,11,11,13,13,15,15-hexadecamethyl.

The compounds identified have interesting biologically relevant properties, including Methoxyphenyl-Oxime, which acts as an antimicrobial agent [33,34], 2-ethylacridine as an antioxidant and antitumor agent [35], Cyclotrisiloxane hexamethyl as an antibacterial, anti-inflammatory and anticancer agent [35], octasiloxane, 1,1,3,3,5,5,7,7,9,9, 11,11,11,15,15-hexadecamethyl has been reported for its antimicrobial properties [36].

The findings of this study thus indicate that the discovered chemical components may be the bioactive components that give *H. aspersa* Müller slime its effectiveness. The above-mentioned chemicals make this slime suitable for usage in a range of medicinal applications.

### 3.2. Mineral analysis

The total ash concentration measured after incineration was 1.09 ± 0.00%, showing that *H. aspersa* Müller contains more minerals. This is an indication of the rich minerals contents that are beneficial.

The Table 3 below contains the findings about the mineral composition of *H. aspersa* Müller slime. High calcium (Ca) concentrations (9.964 ± 0.033 mg.100 g^−1^) were found in *H. aspersa* Müller slime, which also included high levels of phosphorus (P) (6.897 ± 0.041 mg.100 g^−1^) and potassium (K) (4.176 ± 0.037 mg.100 g^−1^). Magnesium (Mg) was detected in the slime in considerable amounts (1.943 ± 0.041 mg.100 g^−1^), although sodium (Na), iron (Fe), and zinc (Zn) were all present in negligible amounts (0.059 ± 0.001 mg.100 g^−1^, 0.102 ± 0.001 mg.100 g^−1^, and 0.044 ± 0.002 mg.100 g^−1^, respectively).

Although they do not work separately, the roles of these minerals are distinct [37].

Sufficient evidence is available in the literature to indicate the effect of mineral elements such as manganese, zinc and copper are involved in tissue, cellular, and subcellular functions, muscle contractions, membrane potential regulation, mitochondrial activity, and enzymatic reactions [38], Grela et al. [39] reported that antioxidant activity has been attributed to mineral components such as copper, manganese, and iron. Pandya [40] revealed that low doses of trace elements such as Cu and Zn inhibit the production of oxidative free radicals. A recent study showed that high antioxidant capacity was correlated with and explained by mineral richness [41], while Phan et al. [42] confirmed that Zn acts as an antibacterial agent.

### 3.3. Antibacterial assay

#### 3.3.1. Disc inhibitory assay

In this study, we found that the mucus of *H. aspersa* Müller also has antibacterial activity. It appears that *E. coli* is susceptible to three antibiotics, as shown by the antibiogram. These include cefotamine sodium, netilmicin, and tetracycline. Antibiotics perform well against *S. aureus* include erythromycin, netilmicin, and tetracycline. *P. aeruginosa*, in contrast hand, has a susceptibility to the antibiotics ampicilline, piperacillin, and tetracycline. Furthermore, *L. monocytogene*, is inhibited by just ampicillin and tetracycline (Table 4).

According to Table 5, we observe that the different concentrations of *H. aspersa* Müller slime used to inhibit pathogenic bacteria have a significant effect that depends on the concentration. We also note that a concentration of 100 μL is the most effective concentration against pathogens. Indeed, it showed an inhibition diameter of 30 mm against *E. coli* (ATCC 35218), while a zone of 23 mm appeared in the boxes containing *P. aeruginosa*. In contrast, similar diameters were observed against *L. monocytogenes* (ATCC 7644) and *S. aureus* (ATCC 29213) (11.1 mm and 12.3 mm, respectively).

These results are comparable to those of Ulagesan and Kim [15], who noted an inhibition diameter of 15.5 mm against *S. aureus* with a concentration of 100 μg·mL^−1^ of proteins extracted from snail slime (*Achatina fulica*), while it was 14.6 mm and 15.83 mm using proteins from *C. bistrialis* and *P. globosa*, respectively. However, none of these species inhibited the growth of *P. aeruginosa*. Another study showed that proteins extracted from the mantle mucus of *Lissachatina fulica* demonstrated the highest antibacterial activity against *E. coli* and *S. aureus* [43].

Furthermore, according to the resistance profile of the tested bacteria, we would like to note that the concentration of snail slime (*H. aspersa* Müller) used was better than most of the antibiotic families used.

We can say that the secreted snail slime presents a composition of biomolecules with antioxidant and antibacterial activities [10,43–45]. Various compounds in snail slime require further characterization and elucidation of their functions in the animal and their appropriate use in various applications.

#### 3.3.2. Determination of the minimum inhibitory concentration (MIC)

**Fig. 2:**
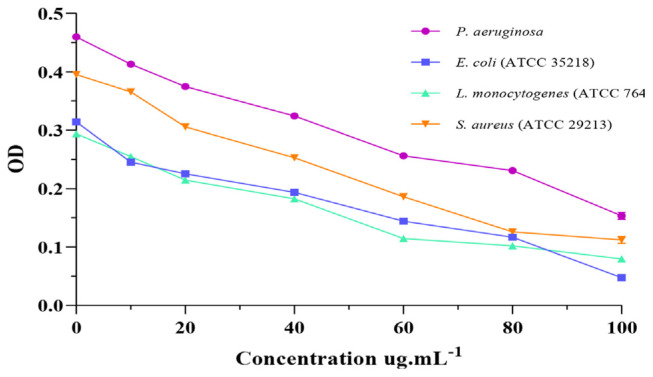
Antibacterial activity of H. aspersa Müller slime.

MIC is the minimum antibacterial concentration required to kill certain bacteria [46]. *H. aspersa* Müller slime exhibited antibacterial activity against all four bacterial strains tested with (Fig. 2). Overall, the findings clearly showed that *H. aspersa* Müller slime had stronger antibacterial activity.

These findings demonstrate that the minimum inhibitory concentration (MIC) for *E. coli* was 10 μg·mL^−1^, the MIC for *L. monocytogenes* was 40 μg·mL^−1^, *P. aeruginosa* and *S. aureus* were stopped at a minimum concentration of 20 μg·mL^−1^.

Our findings are in accordance with the literature on the antibacterial activity of *H. aspersa* Müller slime, according to a comparison of our findings with those of previous investigations.

EL-Zawawy and Mona [47] showed that slime from *H. aspersa* Müller and *Eremina desertorum* had an antibacterial effect against *P. aeruginosa* with MICs of 15 and 7 μg·mL^−1^, *E. coli* 20 and 5 μg·mL^−1^, *S. aureus* 15 and 5 μg·mL^−1^, respectively. In another study, *L. fulica* slime showed an antibacterial effect against *S. aureus*, *S. epidermidis* and *Corynebacterium* spp. with MIC values of 12.5, 25 and 25 μg·mL^−1^, respectively, as well as the MIC values of the in vitro antibacterial activities of *Pomacea canaliculata* slime against *S. aureus*, *S. epidermidis* and *Corynebacterium* spp. being above 50 [48].

According to several investigations, the mucus of *L. fulica* and *H. aspersa* displayed antibacterial activity against diverse species of bacteria and fungi [6,49–52]. In addition, several antimicrobial peptides from the mucus of *L. fulica* and *H. aspersa* have been studied [53–55]. Crude protein isolated from six different species of snails has recently demonstrated antibacterial efficacy against certain bacteria and fungi [15].

### 3.4. Effect of H. aspersa Müller slime on wound area

In the current investigation, in vivo experiments were performed to investigate the healing properties of *H. aspersa* Müller slime.

**Fig. 3:**
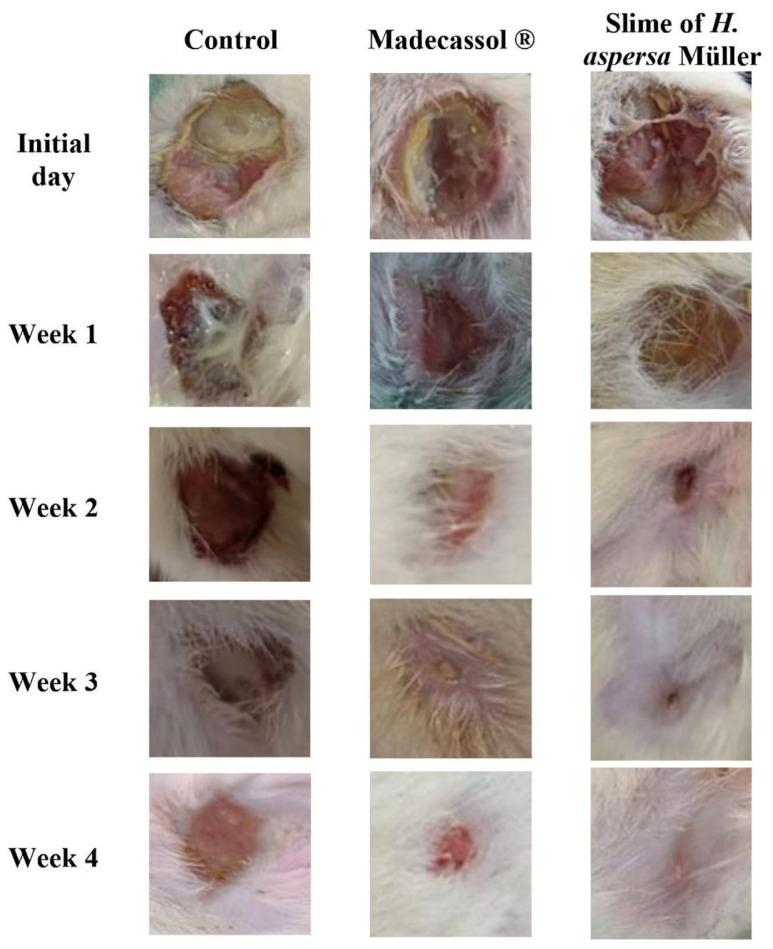
Photos depicting the effect of H. aspersa Müller slime on the wound-healing model at different stages of the study.

The findings of healing activity using the incision wound model are shown in the Table 6 and Fig. 3. The control, *H. aspersa* Müller slime therapy, and standard group healing percentages are represented at 0, 7, 14, 21, and 28 days.

In order to restore the morphological and functional integrity of the epithelial tissues and ensure barrier function, wound healing is a complicated physiological process that involves tissue remodeling and repair [56]. Re-epithelialization and tissue granulation are crucial throughout the healing process [57]. Similar to the re-epithelialization procedure, the time frame in which the barrier function is recovered is crucial [25]. As a result, crucial metrics for measuring the effectiveness of healing include wound contraction, epithelialization, and granulation [58].

In this study, wounds treated with *H. aspersa* Müller slime had a significantly greater capacity for contraction than the standard ointment-treated group, the latter being superior to the control group (p ≤ 0.05) (Table 6).

Both groups hastened wound healing and displayed greater results than the control group by day 14, when wound evaluation revealed that 64% of the wounds in the group treated with *H. aspersa* Müller slime had healed, as opposed to group II, where this proportion was only discovered as early as 21 days. *H. aspersa* Müller slime had a healing effect that was equivalent to that of common, over-the-counter ointment.

In the present study, we demonstrated that *H. aspersa* Müller snail slime also displayed antibacterial action and healing activity, which is consistent with its chemical makeup.

## 4. Conclusion

This study examined the antibacterial, wound-healing properties, chemical and mineral composition of *H. aspersa* Müller slime. According to the study’s findings, this slime exhibits a variety chemical composition of bioactive compounds, a respectable level of minerals, and a sizable amount of antibacterial and wound-healing activity. Due to the presence of bioactive chemicals, it can be used for future clinical and commercial studies, and can be considered as an adjuvant therapy for skin wounds. The biological activities of *H. aspersa* Müller slime suggest that it has positive effects for both medicinal and aesthetic uses. In conclusion, the data reported here might be a useful resource for future study into the use of novel natural compounds to fight microbial diseases.

## Figures and Tables

**Table 1:** Used antibiotics and their families.

Antibiotics	Antibiotic family
Ampicilline (10 μg)	Beta - Lactame
Cefuroxime sodium (30 μg)	Cephalosporine
Cefotamine sodium (30 μg)	Beta - Lactames 3 rd Generation
Erythromycine (25 μg)	Macrolides
Netilmicin (30 μg)	Aminoside
Tetracycline (30 μg)	Tetracycline
Piperacilline (100 μg)	Beta - Lactame

**Table 2:** Chemical composition of H. aspersa Müller slime.

RT	Compounds	Area %
02.342	(Z)-2-heptène (**C_5_H_9_N**)	00.228
03.191	2-éthylacridine (**C_15_H_13_N**)	00.043
04.742	4H-Thiopyran-4-one, tétrahydro-, 1,1-dioxyde (**C_5_H_8_O_3_S**)	00.208
05.098	Thiophène, 3-(décyloxy)tétrahydro-, 1,1-dioxyde (**C_12_H_24_O_3_S**)	00.667
05.507	Méthyltris(triméthylsiloxy)silane (**C_10_H_30_O_3_Si**)	00.295
05.771	Methoxyphenyl-Oxime (**C_8_H_9_NO_2_**)	02.423
07.155	1-Butyl-2,4,6-trimethyl benzene (**C_14_H_20_OS**)	00.093
08.798	Auramine (**C_17_H_22_C_l_N_3_**)	00.414
08.963	N-(trifluoroacétyl)-N,O,O′,O″-tetrakis(triméthylsilyl)norépinéphrine (**C_19_H_34_F_3_NO_4_Si**)	01.367
09.969	3-isopropoxy-1,1,1,5,5,5-hexaméthyl l-3-(triméthylsiloxy)trisiloxane (**C_12_H_34_O_4_Si_4_**)	00.338
11.100	Cyclotrisiloxane, hexaméthyl (**C_6_H_18_O_3_Si_3_**)	47.303
13.476	Mercaptoéthanol (**HOCH_2_CH_2_SH**)	01.098
14.498	N-(2-acétylcyclopentylidène)cyclohexylamine (**C_13_H_21_NO**)	03.057
14.645	Octasiloxane, 1,1,3,3,5,5,7,7,9,9,11,11,13,13,15,15-hexadécaméthyl (**C_16_H_50_O_7_Si_8_**)	04.325
15.560	6-chloro-4-phényl-2-propylquinoline (**C_18_H_16_C_l_N**)	03.117
15.670	10-methylnonadecane (**C_20_H**)	02.295
16.446	Tetradecanal (**C_14_H_28_O**)	00.755
18.025	Furan, 2 isobutenyl-4-vinyl (**C_10_H_10_O**)	00.524
19.233	1,2- Benzene dicarboxilic acid (**C_8_H_6_O**)	11.447
21.960	Cyclohexa-2,5-diène-1,4-dione, 2-méthyl-5-(4-morpholinyl) (**C_11_H_13_NO**)	03.601
	**Not identified**	16.402

**Table 3:** Mineral content of H. aspersa Müller slime.

Elements	Quantity (mg.100g^−1^)
Ca	9.96 ± 0.03
Fe	0.06 ± 0.00
K	4.18 ± 0.04
Mg	1.94 ± 0.04
Na	0.04 ± 0.00
Zn	0.10 ± 0.00
P	6.90 ± 0.04

**Table 4:** Comparison between the effects of antibiotics on pathogenic bacteria in vitro.

Antibiotics	*P. aeruginosa*		*E. coli* (ATCC 35218)		*L. monocytogenes* (ATCC 7644)		*S. aureus* (ATCC 29213)	
			
	ZI (mm)	Pr	ZI (mm)	Pr	ZI (mm)	Pr	ZI (mm)	Pr
AMP	00.00 ± 0.00^a^	R	00.00 ± 0.00^a^	R	00.00 ± 0.00^a^	R	00.00 ± 0.00^a^	R
COF	09.60 ± 0.12 ^b^	S	17.00 ± 0.00^c^	S	10.00 ± 0.09 ^b^	S	00.00 ± 0.00^a^	R
CUS	10.00 ± 1.02 ^b^	S	00.00 ± 0.00^a^	R	28.00 ± 0.16 ^d^	ES	00.00 ± 0.00^a^	R
ETM	19.00 ± 0.24 ^d^	S	00.00 ± 0.00^a^	R	25.00 ± 0.05 ^cd^	ES	20.00 ± 0.00 ^bc^	S
NM	19.67 ± 0.10 ^de^	S	13.50 ± 0.00 ^b^	S	30.00 ± 0.37 ^de^	ES	18.00 ± 0.00 ^b^	S
PRP	00.00 ± 0.00^a^	R	00.00 ± 0.00^a^	R	17.00 ± 0.20 ^bc^	S	00.00 ± 0.00^a^	R
TC	00.00 ± 0.00^a^	R	12.00 ± 0.00 ^b^	S	00.00 ± 0.00^a^	R	15.00 ± 0.00 ^b^	S

Means in the same column with the same letter do not differ significantly from each other at the 5% level of significance according to the Duncan’s test.

ZI: Zone of Inhibition; Pr: Profil; R: Resistant; S: Sensitive; ES: Extremely Sensitive; ER: Extremely Resistant.

**Table 5:** Average diameter of pathogen inhibition zones generated by H. aspersa Müller slime.

Concentration of snail mucus (μg.mL^−1^)	*P. aeruginosa*	*E. coli* (ATCC 35218)	*L. monocytogenes* (ATCC 7644)	*S. aureus* (ATCC 29213)
25	10.5 ± 0.00^a^	18.7 ± 0.07 ^ab^	03.6 ± 0.10^a^	09.6 ± 0.00^a^
50	12.2 ± 0.06 ^ab^	21.0 ± 0.20 ^ab^	05.0 ± 0.13^a^	10.0 ± 0.01^a^
75	15.0 ± 0.10 ^b^	28.1 ± 0.14 ^b^	09.4 ± 0.03 ^ab^	11.9 ± 0.12 ^ab^
100	23.1 ± 0.21^c^	30.0 ± 0.32 ^bc^	11.1 ± 0.00 ^b^	12.3 ± 0.18 ^b^

Means in the same column with the same letter do not differ significantly from each other at the 5% level of significance according to the Duncan’s test.

**Table 6:** Effect of H. aspersa Müller on percentage (%) wound closure.

	Control	Madecassol ®	Slime of *H. aspersa* Müller
Initial day	00.00 ± 0.00	00.00 ± 0.00	00.00 ± 0.00
Week 1	14.57 ± 3.59^a^	23.16 ± 2.99 ^b^	39.81 ± 5.43^c^
Week 2	19.01 ± 4.71^a^	40.01 ± 7.24 ^b^	64.33 ± 4.67^c^
Week 3	38.09 ± 6.52^a^	58.44 ± 6.31 ^b^	76.06 ± 8.30^c^
Week 4	48.23 ± 4.23^a^	81.14 ± 3.24 ^b^	91.73 ± 3.05^c^

Values are expressed as mean ± SD (n = 5). All columns are significant using ANOVA.

P ≤ 0.05 when compared to control.

## Data Availability

No new data was generated for this review.
